# Physical and Mental Health of Children with Developmental Coordination Disorder

**DOI:** 10.3389/fpubh.2016.00224

**Published:** 2016-10-24

**Authors:** Priscila Caçola

**Affiliations:** ^1^Department of Kinesiology, University of Texas at Arlington, Arlington, TX, USA

**Keywords:** developmental coordination disorder, physical health, mental health, motor skills, children

## Abstract

Developmental coordination disorder (DCD) is a neurodevelopmental condition characterized by poor motor proficiency that interferes with an individual’s activities of daily living. These problems in motor coordination are prevalent despite children’s intelligence levels. Common symptoms include marked delays in achieving motor milestones and clumsiness, typically associated with poor balance, coordination, and especially handwriting skills. Currently, DCD is said to impact about 2–7% of school-age children. More importantly, DCD is considered to be one of the major health problems among school-aged children worldwide, with unique consequences to physical and mental health. Because these children and adolescents often experience difficulties participating in typical childhood activities (e.g., riding a bike), they tend to be more sedentary, more overweight/obese, at a higher risk for coronary vascular disease, and have lower cardiorespiratory and physical fitness than their typically developing peers. From another perspective, the motor difficulties have also been linked to an increased risk for mental health issues, such as higher anxiety and depression. The understanding of the health consequences associated with DCD offers practical applications for the understanding of the mechanisms and intervention protocols that can improve the consequences of this condition. In this review, I will explore such consequences and provide evidence for the implementation of interventions that focus on improving physical and mental health in this population.

## Developmental Coordination Disorder

Developmental coordination disorder (DCD) is defined as a neurodevelopmental condition characterized by poor motor proficiency that interferes with an individual’s activities of daily living (1). This disorder defines children who, for no medical reasons, fail to acquire adequate motor skills despite their intelligence levels (2). The movements of children with DCD are often described as “clumsy” and “uncoordinated,” and frequently lead to performance difficulties that typically developing (TD) children perform easily. Children with DCD are not just low in athletic ability; they struggle to perform the everyday activities that most of us take for granted – zipping a knapsack, tying shoes, using scissors, or buttering bread (3). Other general difficulties commonly associated with DCD include poor fine and gross motor control, speech fluency, abnormal muscle tone (hypo/hypertonia), poor body awareness, and gross motor sequencing. Those general complications can be observed when children with DCD attempt to plan a motor task, organize movements, perform a coordinated action, and adjust movements when demands change, such as moving fast to catch a ball (4).

Developmental coordination disorder has been described as a “hidden problem,” (5) with an estimated prevalence as high as 10% in school-aged children. In general, estimates of 2–7% are more likely (6), implying that most school classes have at least one affected child. Estimates suggest that DCD affects four times more males than females, and children born prematurely and/or with extremely low birth weights are at a significantly increased risk of demonstrating this condition (7). Interestingly, these difficulties associated with motor learning and control can last well into adolescence and adulthood (8, 9).

Children with DCD are often diagnosed with other developmental disorders. The most common disorder is attention deficit hyperactivity disorder (ADHD), with 50% of the population showing both disorders. Dyslexia is also a relatively common comorbidity, as well as learning disabilities, speech/language impairment, and most recently, autism spectrum disorders (ASD). Most likely, these diagnoses come before the DCD evaluation, and most of the interventions focus on the comorbidity first. In addition, only ~25% of children with DCD are referred and diagnosed before starting school. The remaining 75% are referred during the first few years in primary school. Presentation at this age includes persistence of the problems noted in the preschool years, such as slow, immature, and laborious handwriting and difficulties in copying from the blackboard. Handwriting problems are readily apparent to classroom teachers, but might be only the tip of the iceberg for children who have significant DCD, indicating that other motor coordination difficulties are present (10). This marked impairment in the development of motor coordination is the first item on the assessment criteria created by the Diagnostic and Statistical Manual of Mental Disorders, Fifth Edition (DSM-5). In this document, DCD is characterized as a Neurodevelopmental Disorder and has four items that must be followed for a correct diagnosis:

1. Acquisition and execution of coordinated motor skills are below what would be expected at a given chronologic age and opportunity for skill learning and use; difficulties are manifested as clumsiness (e.g., dropping or bumping into objects) and as slowness and inaccuracy of performance of motor skills (e.g., catching an object, using scissors, handwriting, riding a bike, or participating in sports);
2. The motor skills deficit significantly or persistently interferes with activities of daily living appropriate to the chronologic age (e.g., self-care and self-maintenance) and impacts academic/school productivity, prevocational and vocational activities, leisure, and play;
3. The onset of symptoms is in the early developmental period;
4. The motor skills deficits cannot be better explained by intellectual disability or visual impairment and are not attributable to a neurologic condition affecting movement (e.g., cerebral palsy, muscular dystrophy, or a degenerative disorder).

## Consequences of DCD

Developmental coordination disorder is considered one of the major health problems among school-aged children worldwide (11), with the outcomes often extending beyond the motor domain to include secondary mental and physical health issues. Most specialists agree that these consequences are the biggest issue when it comes to DCD. While it is possible to remediate some of the motor skill problems in childhood, the mental and physical outcomes can significantly compromise quality of life and health of this population for their entire life. An overview of these outcomes is presented below.

## Mental Health in DCD

Recently, robust evidence has been added to the notion that children with DCD have an increased risk for mental health difficulties (12, 13). Teachers report that school-aged children with DCD have fewer friends and are more socially isolated than their peers (14, 15), and tend to report lower self-esteem, possibly because of the fewer social contacts and friendships (15, 16). The feelings of inadequacy accompanying poor motor coordination may be constantly reinforced through interaction with peers in school (17).

Several research studies have shown that children with DCD have lower levels of participation in physical activities than their peers without DCD in recess and are less likely to engage in both structured and unstructured activities when compared to TD children (18, 19). It is likely that children with DCD frequently withdraw from physical activities due to poor motor coordination and low perceived competence in sports (20). Children with DCD often face frustration engaging in self-care activities (e.g., dressing), school-based activities (e.g., writing), and have less confidence in their ability to play with other children, mainly due to their motor coordination problems (21). As a result of repeatedly being unable to master daily activities, many children with DCD experience a chronic sense of failure that reduces their willingness to participate in physical activities and trying novel tasks (22). Many children with DCD also report lower levels of enjoyment in free play activities, physical education classes, or organized sports (18, 23, 24).

Empirical evidence directly examining the co-occurrence of motor coordination difficulties and depression is growing (3, 25–27). A 3-year longitudinal in which children identified with probable DCD at age 7 were reassessed for mental health difficulties at age 10 reported that children at risk for DCD were significantly more likely to develop mental health problems relative to their peers (12). Even more interesting, this study found that several factors mediated the connection between DCD and subsequent mental health problems. Specifically, those children with probable DCD who had higher verbal intelligence, higher self-esteem, stronger academic performance, good social communication skills, and an absence of bullying were less likely to develop mental health difficulties over time (12). Children with DCD also have been shown to have higher levels of anxiety than children without the disorder (28–30). A recent large study has reported on more symptoms of anxiety and also depression, but only in a population of children with DCD combined with ADHD (31). Interestingly, this was true for both children and parents. This finding leads to the belief that the level of mental health and socioemotional functioning in those with DCD might also be associated with the degree and number of other comorbidities. These problems may be common problems across neurodevelopmental disabilities, and issues can be compounded beyond the lack of engagement in movement settings shown in children with DCD.

In relation to age, a recent systematic literature review found that differences in groups of TD and children with DCD start to emerge between 8–10 and 12–14 years of age – preschool children did not experience or perceive any significant difference (32). It also is possible that the population with DCD may be overrepresented within high school dropouts (33) and adult mental health clinics (30, 34). It is apparent that in late childhood and adolescence, the emotional impact of DCD may be more severe than the primary motor difficulties that are experienced (35). Thus, as individuals with DCD age, the presentation of difficulties may begin to include more psychosocial issues that are more than likely to affect one’s quality of life. For example, adults with a DCD reported significantly lower levels of quality life satisfaction in all domains when compared to TD adults (35). They also report significantly more symptoms of depression, state, and trait anxiety than their peers (36). Retrospectively, adults with DCD have described the anxiousness that they felt about their movement problems in settings, such as physical education and recess (37).

Fortunately, there is recent evidence that it is possible to improve psychological well-being in this population. A pilot study exploring whether two group intervention programs improved several psychological variables (anxiety, adequacy, and predilection for physical activity; participation, preferences, and enjoyment for activities) and motor skills from the perspective of a child with DCD as well as parental perceptions of motor skills, rate of function, and strengths and difficulties (38). The programs were unique in characteristics: Program A focused on task-oriented activities in a large group involving motor skill training and collaboration and cooperation among children, and Program B was composed of three groups with a direct goal-oriented approach for training of skills chosen by the children. Results indicated that children improved motor skills after both programs but showed distinct results in regards to other variables – after Program A, children showed higher anxiety and lower levels of enjoyment, even though parents detected an improvement in rate of function and a decrease in peer problems. With Program B, children decreased anxiety levels and parents noted a higher control of movement of their children. This study establishes a new concept in interventions for children with DCD – while it is important to improve motor skills, most researchers and therapists in the field will agree that prevention and treatment of mental health difficulties should be a key element of intervention for children with DCD (15). Obviously, more studies should be conducted, but an identification of factors that may be modifiable and that can be targeted through intervention is likely to be a critical component of addressing the long-term mental health outcomes of children with DCD (31).

## Physical Health in DCD

Several studies have also identified that children with DCD are physically inactive and less fit when compared to their TD peers (1, 39–42). According to a recent systematic review, 13 of 18 studies examining the relationship between motor proficiency and body composition found that children with poor motor proficiency had significantly higher BMI, waist circumference, and percent body fat compared to their peers (1). The prevalence of overweight and obesity is higher in DCD children, according to a recent systematic literature review. Longitudinal research has also shown poor motor coordination to predict negative changes in body weight, including increased risk of overweight and obesity (43, 44).

Several measures of health-related fitness have been investigated with this population. In a longitudinal study comparing a group of TD children to a group of children with DCD, results indicated that the TD group showed significant long-term changes in BMI and long jump, while the DCD group showed significant increases in BMI values and decreases in flexibility, as measured by the sit and reach task. In general, children with DCD performed worse on the items of flexibility, muscle strength, and muscle endurance after the first year. Compared to age- and gender-matched norms, children with DCD not only were less physically fit but also showed a significant long-term decline in flexibility and abdominal or core strength (45).

Children with lower motor competence also demonstrate significantly poorer performance on important components of physical fitness, such as aerobic and anaerobic endurance and muscular strength, when compared against developing typically peers (46). Several factors that may contribute to poorer fitness in children with DCD have been identified, such as muscular strength, inability to exert maximal force, and variability in rate of power and timing in performing work (47). A recent study has found that children with DCD perform significantly worse than TD children in the five-jump test, triple-hop distance, the modified agility test, and walking distance (48). It also appears that the DCD population also shows lower performance in several physiological measurements, as in poorer lung function (42), earlier exercise fatigue (49), higher blood concentration, heart rate, respiratory exchange ratio, salivary alpha amylase, lower plain threshold (50), and reduced baroreflex sensitivity (51). Overall, it can be easily concluded that indices of health-related physical and cardiorespiratory fitness are lower in children with DCD.

Despite the number of physical health concerns in these children, it appears that it is possible to alleviate some of these issues. As it is been established with mental health, there is recent evidence that it is possible to improve physical health in children with DCD. A recent study explored the effects of a motor skill training on exercise tolerance and cardiorespiratory fitness in this population (48). A DCD training group, DCD control non-training group, and a TD control group were tested for cardiopulmonary exercise test (CPET), pulmonary function testing, and 6-min walking test (6MWT) with an interval of 8 weeks. The training group participated in three sessions per week for 8 weeks. The sessions emphasized improvement of motor skills and fitness. The results indicated that the DCD group improved in maximal power output at anaerobic threshold and at peak level, walking distance, and maximum heart rate. They also reduced perceived exertion, while the other groups did not change any of the measures.

## Concluding Remarks

Developmental coordination disorder is a disorder diagnosed on the basis of poor motor skills. However, the physical and mental consequences of the condition, when present, are substantial and can cause significant long-term outcomes. While it is likely that poor motor coordination results in withdrawal of most, if not all, physical activity settings, it is not known precisely how children with DCD subsequently develop mental health problems like depression and anxiety (24, 36, 52). There are several potential mediators between poor motor coordination and physical/mental health – peer and social relationships, bullying, and self-esteem. We can conclude, in general, that this population is at a significant risk for physical and mental health outcomes, and these risks should be treated seriously and should be the focus of the work with this population, even before symptoms or concerns appear. With that, it is important to recognize that there is also evidence for a lack of differences in children with DCD and TD children in feelings of social acceptance and general self-worth (32) – while this may be due to sample bias and/or other confounding factors, it is important to highlight that some children with DCD may never have mental health problems. However, it is probably best to treat all the DCD population as “high-risk,” and work on prevention of any future health problems.

It is been well-documented that interventions based on everyday motor skills have been effective for the DCD population (53). These interventions focus on findings and implementing strategies that facilitate the accomplishment of motor tasks that are otherwise difficult (e.g., handwriting, tying shoes, etc.). With the latest findings regarding the physical and mental health outcomes, it is imperative that interventions are employed to enhance both physical and mental health in everyday life. Ideally, these interventions would be holistic and focus on prevention of these consequences. Missiuna and Campbell (31) reinforce that a focus on interventions that may be preventative is particularly important in light of the mounting evidence that DCD is a chronic condition that cannot be “fixed” and will persist into adulthood (37, 54, 55).

As these types of interventions might be challenging for professionals, it might be important to start with promoting models that analyze relationships among all factors that place children with DCD at risk for psychological problems in the first place. For example, Cairney et al. (52) explores the environmental stress hypothesis, which suggests that DCD may be a primary stressor leading to secondary stressors, potentially leading to problems such as depression and anxiety. As physical and mental health follow parallel paths, it is important to explore models that account for both. Interventions that account for the factors mediating poor motor coordination and its consequences should be a priority – and as such would need the involvement of several professionals at different levels and expertise. The program Partnering for Change (56) is one such intervention that is currently being tested. While it is complex, and perhaps even overwhelming for health and education professionals to see DCD as more than a motor coordination problem and to focus on prevention of the consequences associated with it, we believe that studies, such as the ones by Caçola et al. (38) and Farhat et al. (48), give some good indications that it is certainly worth trying.

An important consideration in all the research and intervention for children with DCD is causality. Establishing causality will only be possible with long-term studies that map trajectories (31) and monitor potential mediators and outcomes over time. The evidence, currently, suggests that DCD is the primary stressor. For example, when it comes to physical health, DCD is typically diagnosed before signs of overweight status, so it is assumed to be the cause of high body fat. However, little is known about the relationship between weight, DCD, and its comorbidities. Research also shows that obesity status is higher in populations with ASD and ADHD, therefore more research is needed to explore the interaction between weight and these comorbidities with DCD.

Obviously, there are many limitations in the studies that looked at physical and mental health of children with DCD. Further research is needed to establish several parameters of this disorder. The issue of comorbidities needs to be explored in detail. In addition, normal difficulties that happen in adolescence, a period of high risk for both the emergence of neuropsychological problems as well as an increase in obesity and decrease in physical activity, should be ruled out. Nonetheless, much of the evidence is leading to the fact that individuals with DCD show consistent and early emergence of mental and physical health problems, which can greatly impact healthcare, in both costs for the government and wellness of the patient, especially long term.

## Author Contributions

The author confirms being the sole contributor of this work and approved it for publication.

## Conflict of Interest Statement

The author declares that the research was conducted in the absence of any commercial or financial relationships that could be construed as a potential conflict of interest.
